# Draft Genome Sequences of 38 Serratia marcescens Isolates Associated with Acroporid Serratiosis

**DOI:** 10.1128/MRA.00194-19

**Published:** 2019-04-04

**Authors:** Nicole C. Elledge, Ron I. Eytan, Lee J. Pinnell, Reavelyn Pray, Jessica L. Joyner, John P. Wares, Kathryn P. Sutherland, Erin K. Lipp, Jeffrey W. Turner

**Affiliations:** a Department of Life Sciences, Texas A&M University at Corpus Christi, Corpus Christi, Texas, USA; b Department of Marine Biology, Texas A&M University at Galveston, Galveston, Texas, USA; c Odum School of Ecology, University of Georgia, Athens, Georgia, USA; d Department of Environmental Health Science, University of Georgia, Athens, Georgia, USA; e Department of Genetics, University of Georgia, Athens, Georgia, USA; f Department of Biology, Rollins College, Winter Park, Florida, USA; University of Southern California

## Abstract

Serratia marcescens is a Gram-negative bacterium causally linked to acroporid serratiosis, a form of white pox disease implicated in the decline of elkhorn corals. We report draft genomes of 38 S. marcescens isolates collected from host and nonhost sources. The availability of these genomes will aid future analyses of acroporid serratiosis.

## Abstract (precis)

Serratia marcescens is a Gram-negative bacterium causally linked to acroporid serratiosis, a form of white pox disease implicated in the decline of elkhorn corals. We report draft genomes of 38 S. marcescens isolates collected from host and nonhost sources.

## ANNOUNCEMENT

Serratia marcescens is a widely distributed Gram-negative bacillus within the Enterobacteriaceae family (1). The species has long been recognized as an important pathogen of humans (1, 2), insects (3–5), and plants (6). Two ecotypes (pulsed-field gel electrophoresis [PFGE] types PDL100 and PDR60) were identified as causative agents of acroporid serratiosis (a form of white pox disease) in reef-building Acropora palmata corals (7, 8). Given the ecological importance of *A. palmata*, it is imperative to gain a better understanding of the genetic mechanisms underlying acroporid serratiosis and what sets the PDL100 and PDR60 ecotypes apart from other pathogenic strains.

Thirty-five S. marcescens PDR60 isolates were collected from a range of host (*A. palmata*) and nonhost (Siderastrea siderea and Solenastrea bournoni corals, corallivorous snail Coralliophila abbreviata, and wastewater) sources throughout the Florida Keys National Marine Sanctuary (Table 1) (8–10). The WWI31 isolate (obtained from wastewater influent) was virulent against *A. palmata* but had a novel PFGE pattern (8). The PDL100 isolate was obtained previously from diseased *A. palmata* in 1999 (7), and the ATCC 13880 isolate was obtained by others from pond water in the Czech Republic and deposited to ATCC in 1961. Starting with glycerol stocks, each isolate was streaked onto Trypticase soy agar plates (37°C overnight), and isolated colonies were grown in lysogeny broth (37°C overnight with shaking). Total DNA for Illumina sequencing was isolated using a Qiagen DNeasy blood and tissue kit (Qiagen, Valencia, CA, USA) per the manufacturer’s instructions. Genomic DNA for PacBio sequencing was isolated using a cetyltrimethylammonium bromide protocol (11). Illumina sequencing libraries were prepared using a PCR-free TrueSeq DNA kit (Illumina, San Diego, CA, USA) and sequenced at Hudson Alpha Institute for Biotechnology (Huntsville, AL, USA) using paired-end chemistry with 250-bp (EL1 and EL119), 150-bp (EL1, EL119, and KS10), or 100-bp (remaining 35 isolates) read lengths. Additionally, six isolates (EL1, EL116, EL119, EL41, EL60, and KS10) were also sequenced on three PacBio single-molecule real-time (SMRT) cells at the Interdisciplinary Center for Biotechnology Research (University of Florida, Gainesville, FL, USA). Adapter sequences and low-quality bases were removed from Illumina reads with TrimGalore! version 0.4.0 (options, -paired and -retain_unpaired) (12). The processed reads were assembled *de novo* with Velvet version 1.2.10 (options -scaffolding, no; -exp_cov, 80; -cov_cutoff, 10; -min_contig_lgth, 500) (13) using the k-mer sizes listed in Table 1. Hybrid assemblies were constructed with MaSuRCA version 3.2.1 (options, default) (14) using the mean insert sizes and insert size standard deviations calculated with BWA (15). Assembly metrics were determined using QUAST version 5.0.2 (options, default) (16). All genomes were annotated using the National Center for Biotechnology Information (NCBI) Prokaryotic Genome Annotation Pipeline (PGAP) (17).

**TABLE 1:** Accession numbers, genome assembly metrics, and sources for the 38 S. marcescens isolates^*a*^

Isolate	Accession no.	k-mer size	No. of contigs	*N*_50_ value	G+C content (%)	Genome size (bp)	Coverage (×)	No. of genes	Source
EL1^*b*^	CP027796	77	2	5,201,691	59.46	5,240,588	776	4,887	*S. siderea*
EL116^*b*^	PXZP00000000	51	6	1,493,212	59.47	5,254,956	87	4,958	*A. palmata*
EL119^*b*^	PXZQ00000000	77	3	4,696,493	59.45	5,250,706	712	4,895	*A. palmata*
EL41^*b*^	PXZR00000000	51	7	1,445,005	59.46	5,263,255	81	4,998	Wastewater influent
EL60^*b*^	PXZS00000000	51	12	621,937	59.45	5,223,299	317	4,957	*A. palmata*
KS10^*b*^	CP027798	77	2	5,199,459	59.45	5,238,337	152	4,882	*C. abbreviata*
EL3	RCEP00000000	29	300	43,061	59.34	5,261,854	20	5,110	*S. siderea*
EL6	RCEO00000000	33	386	28,046	59.43	5,272,664	20	5,146	*S. siderea*
EL84	RCEN00000000	25	221	51,500	59.46	5,178,755	17	5,037	*C. abbreviata*
EL85	RCEM00000000	27	319	39,739	59.41	5,216,376	19	5,133	*C. abbreviata*
EL95	RCEL00000000	25	469	24,414	59.39	5,160,448	18	5,238	*A. palmata*
EL96	RCEK00000000	23	576	18,069	59.32	5,209,705	19	5,321	*A. palmata*
EL97	RCEJ00000000	25	737	13,342	59.25	5,163,374	22	5,439	*A. palmata*
EL98	RCEI00000000	29	549	19,346	59.42	5,177,503	24	5,294	*A. palmata*
EL108	RCEG00000000	45	35	681,033	59.46	5,226,639	39	4,912	*S. bournoni*
EL109	RCEH00000000	27	424	26,182	59.36	5,274,065	22	5,209	*S. bournoni*
EL110	RCEF00000000	31	274	39,152	59.43	5,217,630	24	5,075	*S. bournoni*
EL113	RCEE00000000	25	251	52,631	59.45	5,193,348	19	5,064	*A. palmata*
EL114	RCED00000000	31	230	47,281	59.45	5,195,401	19	5,054	*A. palmata*
EL115	RCEC00000000	27	293	42,433	59.38	5,342,905	22	5,108	*A. palmata*
EL117	RCEB00000000	27	524	20,600	59.34	5,169,891	19	5,302	*A. palmata*
EL118	RCEA00000000	23	552	18,973	59.30	5,162,774	20	5,292	*A. palmata*
EL120	RCDZ00000000	23	522	22,383	59.31	5,228,290	21	5,292	*A. palmata*
EL121	RCDY00000000	23	497	20,434	59.31	5,224,848	18	5,264	*A. palmata*
EL122	RCDX00000000	23	546	19,553	59.33	5,210,136	20	5,285	*A. palmata*
KS1	RCDW00000000	31	509	20,965	59.43	5,176,180	29	5,247	*C. abbreviata*
KS5	RCDV00000000	29	493	20,749	59.41	5,189,506	25	5,266	*C. abbreviata*
KS9	RCDU00000000	25	372	29,264	59.40	5,165,351	19	5,152	*C. abbreviata*
KS12	RCDT00000000	31	299	39,028	59.29	5,312,456	21	5,107	*C. abbreviata*
KS16	RCDS00000000	35	262	42,604	59.46	5,193,145	24	5,037	*C. abbreviata*
KS23	RCDR00000000	31	194	57,502	59.60	5,172,913	22	4,921	*C. abbreviata*
KS25	RCDQ00000000	29	213	51,510	59.46	5,207,877	21	5,050	*C. abbreviata*
KS40	RCDP00000000	29	468	22,926	59.44	5,226,754	25	5,236	*C. abbreviata*
KS45	RCDO00000000	29	177	34,539	59.38	5,267,350	22	5,011	*C. abbreviata*
KS65	RCDN00000000	33	301	36,304	59.43	5,211,114	25	5,095	*C. abbreviata*
WWI31	RCDM00000000	27	324	34,866	59.86	5,127,259	16	5,009	Wastewater influent
PDL100^*c*^	RCDL00000000	27	494	22,127	59.20	4,963,330	15	5,089	*A. palmata*
ATCC 13880^*d*^	RWJO00000000	33	661	13,688	59.54	5,044,366	31	5,238	Pond water

a All isolates, except the ATCC isolate, were collected in the Florida Keys National Marine Sanctuary (USA).

b Hybrid assemblies.

c Deposited to ATCC in 2002.

d Deposited to ATCC in 1961.

Table 1 shows summaries of the 38 draft genome assemblies. The availability of these genomes will aid in more comprehensive analyses of S. marcescens and acroporid serratiosis.

### Data availability.

This whole-genome shotgun project has been deposited in GenBank under the accession numbers listed in Table 1. The raw sequence reads were deposited in the Sequence Read Archive under the BioProject accession numbers PRJNA494152 (nonhybrid assemblies) and PRJNA438529 (hybrid assemblies).
